# Motor imagery in autism: a systematic review

**DOI:** 10.3389/fnint.2024.1335694

**Published:** 2024-02-12

**Authors:** Emma Gowen, Eve Edmonds, Ellen Poliakoff

**Affiliations:** Division of Psychology, Communication and Human Neuroscience, Faculty of Biology, Medicine and Health, Manchester Academic Health Science Centre, University of Manchester, Manchester, United Kingdom

**Keywords:** autism, MI, action simulation, motor simulation, motor imagery

## Abstract

**Introduction:**

Motor Imagery (MI) is when an individual imagines performing an action without physically executing that action and is thought to involve similar neural processes used for execution of physical movement. As motor coordination difficulties are common in autistic individuals it is possible that these may affect MI ability. The aim of this systematic review was to assess the current knowledge around MI ability in autistic individuals.

**Methods:**

A systematic search was conducted for articles published before September 2023, following PRISMA guidance. Search engines were PsycINFO, PubMed, Web of Science, Scopus, Wiley Online Library and PsyArXiv. Inclusion criteria included: (a) Original peer-reviewed and pre-print publications; (b) Autistic and a non-autistic group (c) Implicit or explicit imagery tasks (d) Behavioral, neurophysiological or self-rating measures, (e) Written in the English language. Exclusion criteria were (a) Articles only about MI or autism (b) Articles where the autism data is not presented separately (c) Articles on action observation, recognition or imitation only (d) Review articles. A narrative synthesis of the evidence was conducted.

**Results:**

Sixteen studies across fourteen articles were included. Tasks were divided into implicit (unconscious) or explicit (conscious) MI. The implicit tasks used either hand (6) or body (4) rotation tasks. Explicit tasks consisted of perspective taking tasks (3), a questionnaire (1) and explicit instructions to imagine performing a movement (2). A MI strategy was apparent for the hand rotation task in autistic children, although may have been more challenging. Evidence was mixed and inconclusive for the remaining task types due to the varied range of different tasks and, measures conducted and design limitations. Further limitations included a sex bias toward males and the hand rotation task only being conducted in children.

**Discussion:**

There is currently an incomplete understanding of MI ability in autistic individuals. The field would benefit from a battery of fully described implicit and explicit MI tasks, conducted across the same groups of autistic children and adults. Improved knowledge around MI in autistic individuals is important for understanding whether MI techniques may benefit motor coordination in some autistic people.

## Introduction

Autism is characterized by persistent difficulties in social communication and social interaction across multiple contexts, including social reciprocity, nonverbal communicative behaviors used for social interaction, and skills in developing, maintaining, and understanding relationships (American Psychiatric Association, DSM-5 Task Force, 2013). Motor coordination difficulties, although not highlighted within this description of autism, are a common feature and are increasingly becoming the subject of investigation (Gowen and Hamilton, 2013; Torres and Donnellan, 2015; Bhat, 2021). Motor coordination difficulties experienced by autistic individuals are apparent from childhood and persist into adulthood and may include altered fine motor control and eye-hand coordination, as well as postural instability and general clumsiness (Fournier et al., 2010; Gowen and Hamilton, 2013; Sacrey et al., 2014; Lim et al., 2017; Morrison et al., 2018; Lum et al., 2020; Gowen et al., 2023). Interest has grown in understanding how motor coordination difficulties might impact upon the social aspects of autism. It has been suggested that exclusion, lack of confidence and fatigue, due to increased effort, could all reduce participation in social activities such as sports, social hobbies or play, leading to less experience in engaging socially (Leonard et al., 2014; Hannant et al., 2016; Ohara et al., 2019; Gowen et al., 2023). Another link between motor and social difficulties is simulation. Simulation theory suggests that when an individual observes another person, they will internally simulate that person’s actions using their own motor, cognitive and emotional representations, in order to understand the other person’s actions (Jeannerod, 2001; Gallese, 2003). Motor simulation is thought to underlie a number of processes such as imitation, action prediction and understanding, as well as motor imagery (Wolpert et al., 2003; Rizzolatti and Sinigaglia, 2010; Gallese and Sinigaglia, 2011; Hurst and Boe, 2022). While some previous work has highlighted altered imitation (Wild et al., 2012; Sacrey et al., 2014; Vivanti and Hamilton, 2014; Gowen et al., 2020), action understanding (Vivanti et al., 2011; Todorova et al., 2019; Federici et al., 2020) and action prediction (Gowen et al., 2022) in autistic individuals, there has been less focus on motor imagery (MI). The aim of this systematic review was to assess the current knowledge around MI ability in autistic individuals.

MI is when an individual imagines performing an action without physically executing that action and is thought to involve similar, although not identical processes to those that program and prepare for the execution of physical movement (Jeannerod, 1994; Hardwick et al., 2018; Hurst and Boe, 2022; Frank et al., 2023). Jeannerod and Frak (1999) argue there are both implicit and explicit forms of MI where actions are imagined at the unconscious or conscious level, respectively. MI can also be performed in the visual or kinesthetic modality. Visual MI involves the visualization of an action and activates visual occipital brain areas, whereas kinesthetic MI consists of imagining the sensation of the action and activates motor-related brain areas such as the superior parietal and ventral premotor cortex (Jeannerod, 2001; Sirigu and Duhamel, 2001; Stinear et al., 2006; Guillot et al., 2009). MI can play a role in practicing, learning and improving execution of actions and is often used by athletes and sports players to enhance performance (Mulder et al., 2004; Simonsmeier et al., 2020; Toth et al., 2020). MI practice, particularly when combined with action observation (Vogt et al., 2013; Eaves et al., 2016, 2022) can also be used for rehabilitation of people with Parkinson’s disease (Tamir et al., 2007; Kikuchi et al., 2014; Caligiore et al., 2017; Bek et al., 2021), as well as stroke patients (De Vries and Mulder, 2007; Sun et al., 2016; Binks et al., 2023) leading to improvements in timed motor performance, limb functioning, daily living skills and reduction of freezing of gait. Therefore, MI training could provide a route to improving motor ability in autism, providing autistic individuals are able to perform MI.

MI can be investigated using several methods including behavioral experiments involving laterality judgments (e.g., right or left hand), mental chronometry and perspective taking, as well as self-report questionnaires (McAvinue and Robertson, 2008). Starting with implicit measures, the hand rotation task (Parsons, 1987) is a method regularly used to investigate implicit MI and many variations of the paradigm have been used. The task requires participants to judge the laterality of hands (right or left) that are presented either from the front (palm) or back view and at different orientations (0–360°). This task uses an implicit measure of MI as participants are not directly instructed to use MI, but engage in this technique to solve the orientation based problem. Outcome measures include accuracy and reaction times. Typically, reaction times increase as the angle increases away from neutral, termed the angle or slope effect and is considered evidence that participants solve the task by mentally stimulating the rotation of their own hand to match the orientation of the displayed hand (Parsons, 1994). However, as it is also possible that participants use a non-MI based strategy such as mental (object) rotation of the hand images (Gardner et al., 2013), a key indicator of whether participants use MI in this task is the effect of biomechanical constraints on reaction times. Reaction times tend to be faster for stimuli where the hands are in a medial rotation rather than lateral direction, due to it being physically easier to rotate your hand in a medial rather than lateral direction (Parsons, 1994; Butson et al., 2014). The stimuli presented in these tasks can vary, with studies either using photographs of real hands or line drawings and some studies use whole bodies, termed the Own Body Transformation task (Gardner et al., 2013) or body rotation tasks.

A variation of the hand rotation task consists of the presentation of two hands or body images that are rotated at different angles and participants are asked to indicate whether the hands are the same or different. This task is based on mental object rotation paradigms where participants are presented with 2D or 3D shapes or objects in different orientations and have to judge whether they match the target shape/object (Shepard and Metzler, 1971). It requires participants to use visual imagery to mentally rotate the object and spatial working memory to hold the image representation in their mind (Shepard and Metzler, 1971; Muth et al., 2014). The use of hands or bodies instead of objects opens the possibility that participants use MI instead of object rotation to complete the task. To differentiate between these two strategies, a steeper reaction time slope across the angles for objects compared to hand (palm) or body stimuli (facing toward) has been interpreted as an object rotation versus a more embodied approach, respectively (Parsons, 1987; Zacks and Tversky, 2005; Yu and Zacks, 2010; Steggemann et al., 2011).

For explicit measures of MI, two common questionnaires are the Kinesthetic and Visual Imagery questionnaire (KVIQ-10) (Malouin et al., 2007) and the Movement Imagery Questionnaire (MIQ) (Hall and Martin, 1997; Gregg et al., 2010). These questionnaires ask participants to perform physically, then explicitly imagine the same movement (e.g., elbow flexion) and rate certain aspects about the imagined movement using Likert-type scales. For the KVIQ-10, participants are asked to rate the clarity (visual subscale) and intensity (kinesthetic subscale) of the imagined movement, while for the MIQ they are asked to rate the ease/difficulty of seeing and feeling the imagined movement. While both measure visual and kinesthetic modalities of MI, unlike the KVIQ, the MIQ is self-administered and measures more functional movements.

Mental chronometry tasks compare the durations of imagined versus executed movements with smaller differences in times suggesting greater accuracy and ability in MI (Wong et al., 2013; Williams et al., 2015). For example, participants might be asked to execute a series of timed pointing movements, then asked to imagine performing the same movements, indicating when they have finished. Better MI ability would be represented as closer durations between the executed and imagined actions. Furthermore, the similarity between real and imagined movements has been taken as evidence of the close relationship between MI and action planning (Jeannerod, 2001; Munzert et al., 2009).

Other behavioral paradigms that potentially measure MI include perspective taking experiments, which require a participant to imagine themselves in a certain orientation and/or from the viewpoint of another person and to then judge the laterality or position of other objects. This type of task can be performed using an embodied strategy of imagining oneself in the new orientation or location of the other or it can be performed using a non-embodied approach such as a rule-based strategy, line of sight computation or by mentally rotating the objects in the scene (Michelon and Zacks, 2006; Kessler and Thomson, 2010; Kessler and Wang, 2012). However, even if individuals use the embodied strategy, these tasks do not necessarily require MI as they can simply be performed by visually imagining the viewpoint from the new perspective, without imagining one’s body parts (Ward et al., 2022). Therefore, whether perspective taking tasks investigate MI varies with different studies. For example, MI is more likely in perspective taking studies that involve referencing a body part or an action (e.g., Conson et al., 2015), as opposed to just reporting the location of objects (e.g., visual perspective tasks in Pearson et al., 2016). More generally, MI of whole body movements is less commonly investigated as highlighted by a meta-analysis by Hétu et al. (2013) who analyzed whole body with upper body tasks due to the small number of tasks in the former category. However, as there are no prior systematic reviews on the topic of MI in autism, we wished to provide a comprehensive review that included less “traditional” MI tasks particularly as perspective tasks are of interest to autism researchers. As the involvement of MI in perspective taking tasks is dependent on study design and instructions, the perspective taking studies included in this review will be individually considered, based on the details of the task to decide whether they measure MI and therefore should be included in the synthesis of results.

This systematic review aims to evaluate and bring together the findings of research investigating MI abilities in autistic individuals using the MI tasks described above. As MI relies on similar motor control processes involved in executing actions, it is possible that motor coordination difficulties frequently present in autistic individuals may lead to altered MI. This review is timely as although there are a growing number of studies in this area, it remains unclear if MI is affected in autistic individuals and if it is, what aspects or in what way is MI affected. A systematic review on the topic of autism and MI is yet to exist in the current literature. A more complete understanding of MI in autism can be helpful for advancing knowledge of simulation ability and whether MI could be used in therapies to support autistic motor coordination difficulties.

## Methods

Please see Supplementary Information S1, S2 for PRISMA checklist.

### Search strategy

A systematic search was conducted in January 2022, following the guidance in the PRISMA statement (Page et al., 2021). The search was conducted in January 2022, then again in September 2023 (for the period 2021–2023) seeking research articles published in journals without restriction based on year of publication. The search engines used were those most relevant to psychology - PsycINFO, PubMed, Web of Science, Scopus, Wiley Online Library and the preprint server, PsyArXiv. The search terms used were autis* AND (Motor Imagery OR “action simulation” OR KVIQ OR “motor simulation” OR “hand rotation task” OR “laterality task” OR “visual imagery” OR “mental rotation”). No limits or filters other than excluding textbooks or books were used. These search terms were based on discussion amongst the authors, examination of key terms in relevant papers and initial scoping searches. As the search function was limited for PsyArXiv, each individual search term (apart from autis*) was entered and the preprints screened to check for any relevant articles. The references list of all studies selected for inclusion in the review were also hand-searched.

### Inclusion and exclusion criteria

Inclusion criteria were: (a) Original peer-reviewed publications and pre-print publications; (b) Participants must include a group of individuals with Autism Spectrum Condition (or Asperger’s) and a non-autistic typically developed group; (c) Articles may include other neurodivergent groups such as Developmental Coordination Disorder. If there is no control group, the paper can be included if there is a comparison with norms OR suitable comparison between real and imagined actions (within the autistic participants group); (d) Participants are asked to engage in a task where the main process involved is either implicit or explicit MI (e.g., hand rotation, perspective taking, chronometry, MI questionnaires). This was judged based on the methods used rather than on the specified focus of the authors. For perspective taking, studies were required to involve explicit instruction for participants to imagine themselves in the position of the character and that the character displayed gestures or movement that could encourage embodiment when identifying objects or performing the task from the required perspective; (e) Measures can involve behavioral (reaction time, accuracy), neurophysiological (e.g., fMRI, EEG) or self-rating measures (e.g., KVIQ, MIQ) to rate their MI; (f) Written in the English language.

Exclusion criteria were (a) Articles only about MI or only about autism; (b) Articles about MI in other conditions and autism, but where the autism data is not presented separately from other conditions; (c) Articles on action observation, action recognition or imitation only where no MI tasks are used. Although spontaneous imagery can occur during action observation (Vogt et al., 2013), it was decided that these tasks do not generally include a specific measure of imagery; (d) Articles were a review or theoretical analysis.

### Screening

Search results were exported to an excel table and duplicates were manually removed using the Microsoft Excel sort filter. Three authors of the review (EG, EP, EE) independently screened a third each of the titles and abstracts of the papers referring to the inclusion and exclusion criteria. During this process the authors highlighted any articles where there was uncertainty about inclusion/exclusion and the three authors discussed these articles to come to an agreement. For the selected papers to be read in full, the same three authors independently read the articles then discussed their findings to come to an agreement.

### Data extraction and strategy for data synthesis

Two authors (EE, EG) extracted the following information into tables: sample size, sex, age, full scale IQ, stimuli and task details, outcome measures and findings. A narrative synthesis of the evidence was conducted. The papers that were included in the review were separated based on the methods they used to measure MI and their characteristics and findings presented within tables and text to enable a within-study analysis. A cross-study analysis was also conducted, comparing the results, characteristics and sources of bias across the studies to inform understanding of MI in autism.

## Results

### Study selection

**Figure 1:**
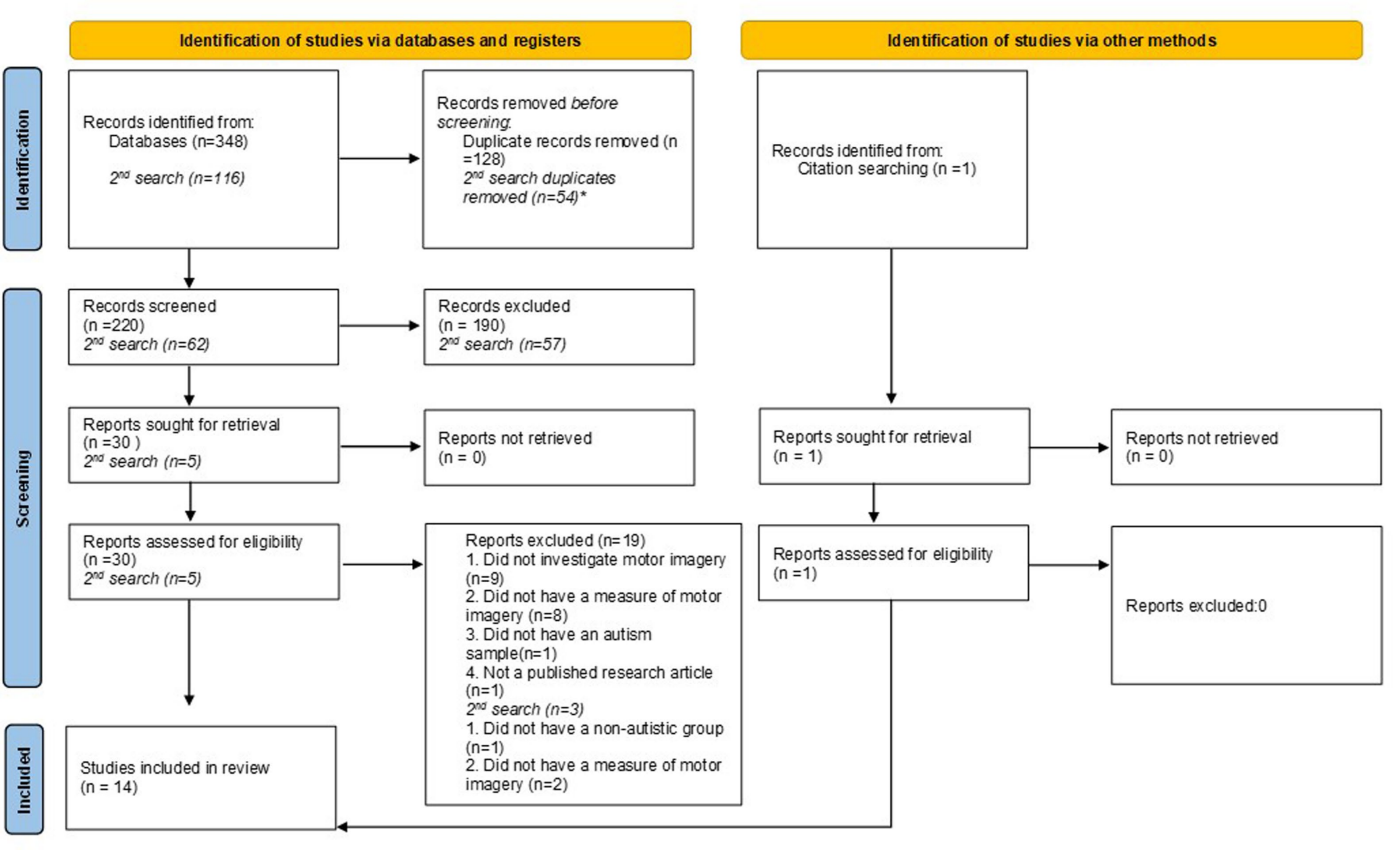
PRISMA 2020 flow diagram for new systematic reviews. Results of the 2nd search in September 2023 are shown separately in italics. *Duplicate removal for 2nd search included 14 articles that were identified in the previous search.

Figure 1 details the process of article selection. From the initial search 348 papers were generated, when duplicates were removed 220 papers were chosen for abstract screening. Thirty papers were selected to be read in full and from these 19 were excluded according to the above criteria. Nine were excluded because they did not investigate MI (they focused on visual imagery or other areas relating to action perception or execution). Eight papers did not have a measure of MI (such as the hand rotation task, KVIQ or any specific instructions to engage in MI). One paper did not have an autism sample and one was a dissertation submission and was not a published research article. From the second search, five articles were selected to be read in full, with one being excluded due to the absence of a non-autistic group and two because they did investigate MI. One article was included following searching the reference lists of the chosen articles, giving a total of 14 articles (16 separate studies) included in the review.

### Quality assessment

The Joanna Briggs Institute (JBI) checklist for case control studies (Moola et al., 2020) was used to evaluate the articles as it was deemed to contain the most relevant items for the types of studies reviewed. However, not all items were relevant and the meaning of some items was modified to fit the types of studies included as detailed in Supplementary material S3. All but one study was rated as acceptable to include apart from one (Xie et al., 2022, hand rotation task), but this was included due this being the first systematic review on the topic and the overall low number of articles (Supplementary material S3).

### Study characteristics

Of the 16 studies included in this review, six used a hand rotation task, four used a body rotation task, three used a perspective taking task, one used the KVIQ and two used explicit instructions to imagine performing a movement. The studies and tasks used in each are outlined in Tables 1–3. The findings from the studies are discussed below and have been grouped into implicit and explicit measures of MI.

**Table 1:** Hand rotation tasks.

Study		Autistic	Non-autistic	Stimuli and task	Findings
Chen et al. (2018)	N(f) Age IQ	22(2) 12.95 ± 1.04 108.48 ± 17.70	22(2) 13.47 ± 1.24 109.64 ± 9.42	3D images of front and back of hands either with spoon or bare hand. Rotated within frontal plane, sagittal plane and transverse plane. 6 orientations (0,60, 120, 180, 240, 300°) Response: right or left hand index finger key press	RT ASC >NT; Significant biomechanical effect in both groups and no group differences (when RT taken into account using biomechanical index) Significant angle effect for both groups Accuracy: No group differences
Conson et al. (2013)	N(f) Age IQ	24(3) 13.4 ± 1.3 100	24(4) 13.3 ± 1.4 99.4	Line drawings of back of hands 4 orientations (0, 90, 180, 270°) Response: index or middle finger key press of right hand	RT: Significant biomechanical effect in non-autistic group only Significant angle effect for both groups Error rate Significant biomechanical effect in non-autistic group only
Conson et al. (2016)	N(f) Age IQ	18(1) 14.6 ± 4.2 110.3 ± 14.7	18(2) 14.8 ± 3.5 104.4 ± 7.5	Full color photographs of front and back of real hands 4 orientations (0, 90, 180, 270°) Response: right or left foot pedal press	RT ASC >NT Significant biomechanical effect in both groups Error rate Larger biomechanical effect for autistic group Significant effect of posture on the biomechanical effect in autistic but not TD for RT and error rate
Johansson et al. (2022)	N(f) Age IQ	14(4) 7.10 82 ± 15.3	17(9) 7.9 106 ± 9.4	Color images of back of hands 4 orientations (0, 90, 180, 270°) Children tested at 7, 8, 9 years. Response: right or left hand button press	RT and error rates Significant biomechanical effect in both groups for 7 and 9 years Absent biomechanical effect for autistic group at 8 years
Soulières et al. (2011)*	N(f) Age IQ	Peak: 14 (2), No-peak: 11(1) Peak:20.6 ± 7.2 No-peak: 21.4 ± 4.6 Peak: 101.7 ± 13.1, No-peak: 102.1 ± 11.5	14(2) 19.4 ± 3.8 103.0 ± 10.5	Line drawing of hands, different gestures 10 orientations (0–180°) Task: Participants presented with 2 stimuli and indicate with a key press whether same/different	RT No group differences Significant angle effect for all groups Accuracy Autistic peak group were significantly more accurate than non-autistic group; no differences between non-autistic and No-peak groups. Significant angle effect for all groups
Xie et al. (2022)	N(f) Age IQ	8 10.8 ± 2.4 –	8 10.5 ± 2.4 –	Not described	RT No group differences Accuracy Non-autistic > autistic

N(f), number of participants, with number of females in brackets. Mean age and IQ are given together with standard deviations. *Note that autistic group was split into groups based on performance of Wechsler block design task with those scoring relatively higher than their overall score being in the Peak group and the rest in the Non-peak group.

**Table 2:** Body rotation tasks.


Study		Autistic	Non-autistic	Stimuli and task	Findings
Pearson et al. (2014)	N(f) Age IQ	18(1) 19.77 ± 4.95 97.61 ± 19.11	18(1) 18.44 ± 3.43 101.55 ± 18.33	Computer generated 3D images of person with one extended arm presented at different angles (0–160°) Egocentric task: Judge whether left/right arm of person is extended Mental rotation task: Judge whether two images are the same/different Response for both tasks: right or left hand index finger key press	Egocentric task: RT greater and accuracy lower for autistic group Significant angle effect for accuracy with no group differences Significant angle effect for RTs for both groups, larger for autistic group (steeper slope) Mental rotation task: Significant angle effect for accuracy with no group differences Significant angle effect for RT, no group differences in slope. Increased RTs for autistic group
Conson et al. (2015)	N(f) Age IQ	22(2) 13.3 ± 1.4 100.2 ± 5.2	22(2) 13.5 ± 1.5 100 ± 3.8	Line drawing of human figure from front or back view in 4 different angles. Judge whether left or right hand marked Response: index or middle finger key press of right hand	Accuracy: No significant angle effect Non-autistic group more accurate for back facing then front facing figures and more accurate than autistic group for back facing. Reaction time Significant angle effect for both groups Non-autistic group faster for back facing then front facing figures, reverse for autistic group; Non-autistic group slower than autistic group for front facing and reverse pattern for back facing.
Pearson et al. (2016)	N(f) Age IQ	30(27) 9.03 ± 2.45 6.55 ± 2.19	30(18) 6.83 ± 1.66 6.68 ± 2.12	Photos of an actor in different postures. Participants point to which one of two photos matches the exemplar photo shown	No group differences in accuracy
Ring et al. (2018)*	N(f) Age IQ	23(3) 38.81 ± 11.82 110 ± 18.7	18(8) 42.12 ± 12.14 110 ± 17.9	Image of man presented as right way up or standing on his head with his front or back to the participant Judge whether disk in left or right hand Response: unspecified	No significant group differences in accuracy or RTs

N(f), number of participants, with number of females in brackets. Mean age and IQ are given together with standard deviations. *Note that authors report that 4 participants did not complete the tasks or were excluded, but the remaining participants were still matched on age, gender and FSIQ. RTs, reaction times.

**Table 3:** Explicit motor imagery tasks.

Study		Autistic	Non-autistic	Stimuli and task	Findings
Gowen et al. (2022)	N(f) Age IQ	20(12) 32.1 ± 6.3 118 ± 12.7	22(11) 28.4 ± 7.5 114 ± 17.6	Questionnaire: KVIQ-20	No significant group differences
David et al. (2010)	N(f) Age IQ	19(8) 36.4 ± 9.3 131 ± 14	15(11) 31.2 ± 6.3 130 ± 10	Perspective taking task Virtual character in front of two objects. Characters gesture to object, facial expression and body orientation changed between trials. Participants required to indicate which object was elevated from perspective of character	No group differences in accuracy or RT
Conson et al. (2015)	N(f) Age IQ	22(2) 13.3 ± 1.4 100.2 ± 5.2	22(2) 13.5 ± 1.5 100 ± 3.8	Perspective taking task Photos of an actor who could be gazing toward/away or grasping/not grasping an object Judge whether the object is on the left/right of the actor from the view point of the actor	Faster RTs and increased number of correct (allocentric) responses when actor grasping object in non-autistic group only
Gauthier et al. (2018)	N(f) Age IQ	26(5) 12.65 ± 3.66 94.33 ± 30.6	38(15) 12.03 ± 3.38 -	Perspective taking task 3D avatar of a tightrope walker Participants instructed to imagine their body in the position of the avatar and lean in the direction he was leaning Movement direction measured	No significant group differences
Piedimonte et al. (2018)	N(f) Age IQ N(f) Age IQ	Adolescents 16(14) 13.8 ± 2.9 102.06 ± 5.22 Adults 12(11) 28.3 ± 4.8 97 ± 16	Adolescents 18(16) 14 ± 2.9 101 ± 2.68 Adults 11(10) 24 ± 2.8 102 ± 1.8	Spatial bimanual task Drawing line with right hand while drawing or imagining drawing a circle with left hand	Significant effect of imagery condition only in non-autistic group
Xie et al. (2022)	N(f) Age IQ	20(4) 10.6 ± 2.69 60 ± 12.57	20(4) 10.88 ± 2.39 61.25 ± 12.76	Recall task Participants given instructions for a series of actions then asked to look at pattern, imagine performing the actions or physically execute the actions Outcome measure of span score: number of actions in the sequence orally recalled	Non-autistic span score: imagery condition>observe pattern condition Autistic span score: imagery condition = observe pattern condition Both groups: execution condition>observe pattern condition

N(f), number of participants, with number of females in brackets. Mean age and IQ are given together with standard deviations.

### Implicit measures of MI

#### Hand rotation tasks

Details of the hand rotation studies can be seen in Table 1 two articles reported that the autistic group displayed a biomechanical effect, but there were differences in the way the two groups performed the task (Conson et al., 2016; Chen et al., 2018). Chen et al. (2018) found no group differences in accuracy and observed a significant slope effect in both autistic and non-autistic children and adolescents, although the former group had slower reaction times (RTs). They also initially observed a larger biomechanical effect in the autistic group. However, when they took into account overall RTs across all the presented angles on the biomechanical effect they found no group differences, confirming that larger RT differences between the medial and lateral orientation in the autistic group was the result of slower overall RTs. A good feature of this study was the inclusion of an object rotation task, which revealed no group differences, suggesting that slower reaction times in the hand rotation task were related to processing the hand rather than general mental rotation and motor coordination difficulties. In addition, Chen et al. also observed a similar biomechanical effect for transitive (hand with spoon) and intransitive (bare hand) stimuli, with both groups showing a larger biomechanical effect for the transitive condition. Conson et al. (2016) asked autistic and non-autistic children and adolescents to perform the hand rotation task with their arms in a posture that either matched or did not match the hand stimulus. Typical individuals show a *posture effect* whereby they are more accurate and show faster RTs when making hand laterality judgments that match their own posture (Shenton et al., 2004; Funk et al., 2005; Ionta and Blanke, 2009). Conson et al. (2016) hypothesized that as covert MI and overt execution become more independent during development, if motor simulation in autistic individuals is not fully effective, body posture would impact upon the ability to perform the task, influencing the biomechanical effect. As with Chen et al. (2018), RTs were slower for the autistic group and there was a significant biomechanical effect for both groups. However, this biomechanical effect was modulated by posture only in the autistic group, so that RTs were faster for postures that matched the hand stimuli, compared to postures that did not match. It is curious that the non-autistic group did not show a significant posture effect as in previous studies (Shenton et al., 2004; Funk et al., 2005; Ionta and Blanke, 2009). Consequently, another interpretation of these results is that the autistic group were behaving as expected, whereas the non-autistic group used a “shallower” form of MI. Overall, the results from these two studies suggest that autistic individuals can perform MI as evidenced by a biomechanical effect, but that this process is more effortful, inefficient or “deeper.”

In contrast to the above studies, Conson et al. (2013) did not find slower RTs or a significant biomechanical effect for RTs, or error rate, in autistic children and adolescents even though it was present in the non-autistic group. There was a significant slope effect in both groups which the authors suggested was due to the autistic group using mental object rotation to rotate the images, but unlike the non-autistic group did not use an embodied approach. Equivalent performance for both groups was found in a letter rotation task, emphasizing intact mental rotation in the autistic group. Chen et al. (2018) suggested that the discrepancy in findings could be due to the use of simpler stimuli reducing the engagement of MI (Dahm et al., 2022): Conson et al. (2013) used line drawing stimuli rather than 3D color images or photos and presented the hands in the back view only as opposed to front and back. This suggestion is supported by the lack of RT differences, suggesting that the autistic group found the task less complex and potentially used a strategy that did not involve MI.

A recent study used the hand rotation task to compare the same autistic and non-autistic children on the hand rotation task at three different age levels – 7, 8, and 9 years (Johansson et al., 2022). The autistic children had longer RTs and in contrast to the non-autistic group, who showed a biomechanical effect at each age point, they only showed a significant biomechanical effect at 7 and 9 years of age. Although this could suggest inconsistent MI, as the authors point out it could also reflect low power as there was a higher error rate for the lateral orientation in the 8 year old autistic group, so more trials would have been removed. In addition, at the start of the experiment, the authors checked whether the children were able to differentiate between a upright right and left hand and 5, 4 and 2 autistic children in the three age groups, respectively, were unable to perform this and therefore continue to the hand rotation task. Although this was a good check to perform, a limitation of this study is the low autistic participant number. The autistic groups also had slower RTs on a number rotation task at each age level, suggesting more general difficulties with mental rotation. Furthermore, the non-autistic group had higher intellectual abilities and a larger number of females. The inclusion of only the backs of hands may also have led to differences in MI between the groups as noted earlier.

As a later adjunct to their explicit imagery task (see later), Xie et al. (2022) conducted a hand rotation task on autistic and non-autistic children and adolescents. As this was a follow up study contained in the articles Supplementary materials, minimal details are given and they reported no group differences in RTs, but less accurate performance for the autistic group. As no biomechanical effect was included, it is difficult to know whether either group used MI and the sample size is small.

One study used the mental object rotation version of the task in autistic and non-autistic adolescents and young adults (Soulières et al., 2011). Participants were presented with two line drawings of a hand gesture rotated at different angles and were required to press a key to indicate whether the gestures were the same or different. In half the trials they were identical and in the other half one of the drawings was the mirror image. In order to successfully complete the task, participants must rotate one hand stimulus to compare whether it is identical to the other. The researchers were interested in understanding how the mental imagery ability of autistic individuals related to other visuospatial skills, such as those used in the Block Design subtest of the Wechsler Intelligence scales. Participants were divided into two groups according to whether they scored more highly (peak group) or not (no-peak group) than the standard scores on the Block Design subtest. Results indicated that the peak group only had higher accuracy then the non-autistic group, although it is unclear from the statistical reporting whether there were differences between the two autistic groups. A significant slope effect was found for all groups. The biomechanical effect was not examined so it is unclear whether participants were using MI to complete the task, although it could be argued that the visual comparison nature of the task, along with it being performed in the same session as other mental imagery tasks using geometrical figures and letters encouraged an object rotation approach.

In summary, repeated findings of an intact angle effect in studies examining hand rotation indicate that mental rotation of hands is intact in autistic individuals and even potentially enhanced in those with better visuospatial skills (Soulières et al., 2011). However, as not all studies measure the biomechanical effect it is unclear whether autistic participants are using a MI or a more general object rotation strategy. Those studies that have examined the biomechanical effect suggest that autistic individuals are using MI, although this seems more challenging compared to non-autistic individuals. Furthermore, autistic individuals may opt for object rotation strategies during simpler tasks where MI is less necessary or useful (Conson et al., 2013). A final important point is that all these studies have been mainly performed on children or adolescent males, with very few female or adult participants.

#### Body rotation tasks

Details of the body rotation studies can be seen in Table 2 and Pearson et al. (2014) explored two body rotation tasks in autistic and non-autistic adolescents and young adults. In one task, termed the egocentric task, participants were asked to view an image of a man and judge the laterality of his extended arm using a keyboard press. For the mental rotation task, they were asked to judge whether two images of the man at different angular rotations, presented at the same time, were the same or different. The authors reasoned that the egocentric task involved participants relating their own body to the image on the screen (i.e., using MI), whereas the mental rotation task involved rotating and comparing the two images without relating to the participant’s own body (see discussion section for further details around this assumption for same/different judgment tasks). Accuracy was lower and RTs higher for the autistic compared to non-autistic group in the egocentric task. Both groups showed a reduction in accuracy and longer RTs with increasing angle and this pattern in RTs was more apparent for the autistic group. For the mental rotation task, accuracy decreased with increasing angle and there were no significant group differences. However, RTs were slower for the autistic group, particularly for larger angles suggesting that they found the task more challenging. Overall, these findings suggest that the autistic group had greater difficulties for both tasks, but this was more apparent for the egocentric task where the presence of a (steeper) slope effect in the autistic group suggests more effortful MI. One confound mentioned by the authors is that as the egocentric task involves a laterality judgment and the mental rotation a same/different judgment, results could have been affected if autistic participants had a greater tendency to confuse right and left, particularly as the two groups were not matched on handedness.

Similar to the mental rotation task above, Pearson et al. (2016) used a variant of the body rotation task where children were shown a picture of a person in a particular posture (e.g., extending an arm) and had to choose which of two further pictures was a match. Although the non-autistic group were significantly younger, they were matched on verbal mental age. As found by Pearson et al. (2014), there were no significant differences in accuracy between the autistic and non-autistic children, although RTs were not reported.

Conson et al. (2015) presented autistic and non-autistic children and adolescents with a line drawing of a person facing toward or away from them, at four different angles, and asked them to judge whether the left or right hand of the person was marked. Participants were explicitly instructed to imagine themselves from the viewpoint of the figure. Non-autistic participants were more accurate and faster when judging back than front facing figures whereas the reverse was true for the autistic participants. This advantage for back facing figures is thought to be due to participants more easily imaging themselves into the position of the body presented, as opposed to either using mental object rotation strategies or needing to initially rotate oneself before superimposing one’s body on the image in front facing images (Kessler and Thomson, 2010; Conson et al., 2015; Dahm et al., 2022). Conson et al. (2015) suggest that the pattern reflects autistic individuals using a non-embodied mental object rotation strategy for both views, which is supported by a jump in RTs between 90 and 180 degree angles in the back facing view for the non-autistic group only. This jump reflects the increased difficulty in rotating oneself to superimpose on the upside-down figure. However, it is difficult to know what strategy the autistic group was using. It is possible that this pattern in the autistic group was due to better object rotation processes for front facing stimuli and altered MI for back facing stimuli, with the jump in RTs being absent because of overall longer RTs. Or it could be that they use a non-embodied strategy for both tasks, which is more efficient for front facing but less efficient for back facing.

Ring et al. (2018) used the “Manikin task” where autistic and non-autistic adults were required to report whether a man on the screen was holding a disk in his left or right hand. The man was presented the right way up or standing on his head with his front or back to the participant. There was no significant difference between the groups in terms of accuracy and RT, although there was a trend approaching significance for the autistic group to be more accurate. A drawback of this study is that as the task was performed as a control task to the main task assessing navigation, there are limited details on the stimulus and results for each orientation.

Summarizing the above studies, it is difficult to interpret whether participants were using MI due to the differences in stimuli and reported outcome measures across studies, as well as a lack of specific MI measures such as the biomechanical effect. Similar to the hand rotation studies, the majority of participants are male. Those studies with more details (Pearson et al., 2014; Conson et al., 2015) suggest that an embodied MI strategy may be more challenging and that mental, object rotation strategies may be favored by autistic participants.

#### Explicit measures of MI

##### Questionnaire studies

Details of the explicit MI studies can be seen in Table 3 and Gowen et al. (2022) used the KVIQ-20 as one of the secondary measures in their study on action prediction. They found no significant differences between autistic and non-autistic adults for either the visual or kinesthetic dimension. Values for visual and kinesthetic components in this study were higher (i.e., more vivid imagery) than previously reported normative values, but the direction of higher visual compared kinesthetic scores was consistent with previous observations (Malouin et al., 2007).

##### Perspective taking tasks

David et al. (2010) asked adult participants to indicate which of two objects was elevated from the viewpoint of a virtual character whose gesture, facial expression and body orientation varied on each trial. For example, the character could be pointing to one object with a neutral expression and body turned toward the object. Participants were explicitly asked to imagine themselves standing in the position of character. There were no group differences in RTs or accuracy.

Using a similar design to David et al. (2010), Conson et al. (2015) asked autistic and non-autistic children and adolescents to observe a photograph of a person sitting at a table with a bottle or glass placed to the left or right of them and indicate which side the bottle was located. They were instructed to make the judgment from either their own perspective, or the perspective of the person. The actor could be gazing toward/away or grasping/not grasping the object. Importantly, it has been previously shown that non-autistic participants imagine themselves from the viewpoint of the figure more frequently when the figure is grasping the object, reflecting embodiment processes. Focusing only on the “other” condition in the non-autistic group, the number of allocentric responses (from the viewpoint of the person in the photo), was significantly higher in the no gaze/no grasp., no gaze/yes grasp and yes gaze/yes grasp condition then the no actor condition. Similarly, RTs were faster in the no gaze/yes grasp and yes gaze/yes grasp conditions than the no actor condition. In contrast allocentric responses and RTs did not differ across conditions for the autistic group suggesting that they do not use an embodiment strategy when asked to take the perspective of the other person. These findings appear to contrast with David et al. (2010), although this earlier study did not compare responses across the different gestures, facial expressions or body orientations. It is will be important for future research to clarify whether autistic individuals are less sensitive to using these cues, rather than there being an impairment of MI *per se*. For example, previous work examining automatic imitation in autistic individuals suggests that while automatic imitation is intact (Cracco et al., 2019), modulation of automatic imitation by top-down cues such as facial expressions or social words is altered in autistic compared to non-autistic individuals (Bird et al., 2007; Forbes et al., 2017; Poliakoff and Gowen, in press).

Gauthier et al. (2018) used imitation to explore perspective taking in autistic children and adolescents. Participants observed a 3D avatar of a tightrope walker from the front or back and were instructed to imagine their body in the position of the avatar and lean in the direction he was leaning. If participants were to imagine themselves from the perspective of the tightrope walker, they should lean in the same direction. For example, if the tight rope walker leans to the right when front facing, the participants should also lean to the right (rather than an egocentric approach of mirroring the movement to the left). Findings revealed no significant differences between the groups, although both groups had high levels of ego centered movements in the front facing condition suggesting that this was challenging for both groups.

Results from relevant perspective tasks are limited and ambiguous, but the study by Conson et al. (2015) is suggestive of autistic children using a strategy that does not involve MI.

##### Spatial bimanual task

Piedimonte et al. (2018) found a significant difference between autistic and non-autistic adults and adolescents on a bimanual task that involved continuously drawing a line with their right hand in three conditions (a) on its own (unimanual condition) (b) while drawing circles with their left hand (bimanual condition) (c) while imagining drawing circles with their left hand (imagery condition). They calculated an Ovalisation Index for each participant, which was used to measure the deviation of the right-hand drawing from a vertical axis. Autistic and non-autistic participants showed a similar coupling effect (significant increase of ovalization index from the unimanual condition) in the bimanual condition. However, in the imagery condition, a significant coupling effect was only found for the non-autistic participants which remained the same when group comparisons were also performed for adults and adolescents separately. The study is interesting as it is one of the first to explicitly ask autistic participants to use MI and directly compares a motor with a MI task, suggesting that imagery is specifically affected. However, participants were not asked about how they completed the task, so it is possible that they did not understand or chose not to use MI. Another limitation is that the Ovalisation Index of the unimanual condition appears to be larger in the autistic compared to non-autistic group, which may have hidden any effects of imagery.

##### Recall task

Xie et al. (2022) examined the effect of MI or execution on the ability to recall a sequence of actions. Autistic and non-autistic children were given a series of instructions involving a sequence of actions on objects (e.g., push the mirror, shake the glove, touch the umbrella) then asked to either look at a pattern, imagine performing the action or perform the action. They then needed to orally recall the sequence of actions. Both groups showed better recall span (higher number of sequences recalled) following physical execution, but only the non-autistic group showed an improvement in recall for the MI condition. This is an interesting study, but as with Piedimonte et al. (2018) it is difficult to know whether the autistic children were unable to perform MI or simply did not attempt it. It would also be interesting to know whether those participants who physically performed the task before the MI condition were better able to engage in MI and whether this was more apparent for the autistic group.

## Discussion

This review aimed to provide an understanding of the current state of knowledge around MI in autism. Focusing on the implicit tasks, there appears to be good evidence of a biomechanical effect in children for hand laterality tasks (Conson et al., 2016; Chen et al., 2018; Johansson et al., 2022), suggesting the presence of MI. Where a biomechanical effect was absent in the autistic group, this seems to be explained by methodological differences such as reduced stimulus richness or limitations in power (Conson et al., 2013; Johansson et al., 2022). Interestingly, all of these hand rotation studies involve children and adolescents, with none being performed with autistic adults. For the body rotation studies that use a laterality judgment, there are mixed results with evidence of differences in processing between the two groups (Pearson et al., 2014; Conson et al., 2015) or no group differences (Ring et al., 2018). Although further studies are required, there does not appear to be a difference in findings according to age. However, the challenge with these tasks is that they do not consistently report the same measures (e.g., front vs. back and angle effect), and without these tasks being able to measure a biomechanical effect it is difficult to interpret how participants are performing the task. Furthermore, when an angle effect is measured, the presence of a steeper slope in autistic individuals (Pearson et al., 2014) could be interpreted as either difficulties in MI processing or “deeper” MI (Kessler and Wang, 2012). The latter fits with the observation of an increased posture effect in the autistic group (Conson et al., 2016).

The presence of implicit MI in the hand rotation task fits with findings of comparable automatic imitation ability in autistic and non-autistic groups (Cracco et al., 2019; Poliakoff and Gowen, in press), both of which are thought to involve motor simulation. However, group differences have been observed for other behaviors that involve motor simulation such as voluntary imitation (Wild et al., 2012; Edwards, 2014; Vivanti and Hamilton, 2014; Gowen et al., 2020), action understanding (Vivanti et al., 2011; Todorova et al., 2019; Federici et al., 2020) and action prediction (Gowen et al., 2022). It may be that in comparison to implicit MI and automatic imitation, these other behaviors require dynamic and more detailed sensory motor processing involving comparison with predictions to produce an accurate percept or action. This greater demand on sensorimotor integration could result in group differences (Gowen et al., 2023).

Three tasks used the same/different judgment variant of the rotation tasks (Soulières et al., 2011; Pearson et al., 2014, 2016). However, for these type of tasks, object rotation strategies appear to be the preferred strategy in typical populations (Zacks and Tversky, 2005; Yu and Zacks, 2010; Steggemann et al., 2011), limiting the conclusions that can be drawn from these studies. In addition, the biomechanical effect tends not to be examined in these tasks, although this is possible to include. Furthermore, none of the studies directly compared the angle effect between hand (palms) or body (facing toward) and object stimuli which has been the previous method in typical groups to differentiate MI from object rotation strategies (Parsons, 1987; Yu and Zacks, 2010; Steggemann et al., 2011). It is interesting to note that autistic and non-autistic groups perform more similarly on this task, suggesting that mental object rotation is similar in both these groups. Indeed, including an object rotation task with all hand and body rotation tasks is valuable for several reasons including distinguishing whether group differences could be due to general mental rotation and motor coordination difficulties, as well as difficulties in left/right judgments. Interestingly, those studies that included both a hand and object rotation task (Conson et al., 2013; Chen et al., 2018) showed that group differences were only present in the former task highlighting that that these group differences were specific to hand processing and unlikely to be caused by the above confounding factors.

A variety of tasks examined explicit MI in autistic children, adolescents and adults. As discussed in the introduction, visual perspective taking tasks do not necessarily involve MI (Ward et al., 2022), so we focused on those tasks that made reference to body parts and were more likely to elicit MI. There are too few studies to make firm conclusions, although the findings of Conson et al. (2015) showing that the responses of the autistic group did not vary when the observed actor was grasping an object suggests that MI was not used. Surprisingly only one study has used a questionnaire measure (Gowen et al., 2022) and found no group differences in their self-rated clarity and intensity of visual and kinesthetic imagery. Of note is that it has been suggested that questionnaires such as the KVIQ could be used to provide a “snapshot” of MI ability as they involve generation of a motor image, which may be most critical for MI ability (Kraeutner et al., 2020). The two studies that compared explicit MI with physical execution (Piedimonte et al., 2018; Xie et al., 2022), both revealed that the autistic group did not engage MI. These explicit tasks would benefit from qualitative input from the participants as in previous work with non-autistic groups (Zacks and Tversky, 2005; Yu and Zacks, 2010; Gardner et al., 2013) to understand what strategies they were using and add context to the findings. There were no clear differences in MI according to age group, although the same tasks would need to be conducted across ages to clarify this. A further consideration for both the reviewed and future explicit MI studies is that a full description of the instructions is provided. For example, Van Caenegem et al. (2022) highlighted substantial underreporting of the instructed modality and perspective which can lead to challenges with replication, understanding and synthesizing of studies, which we would add is particularly important when studying heterogeneous conditions such as autism. Future studies would benefit from following the recently published *Guidelines for Reporting Action Simulation Studies* (Moreno-Verdú et al., 2023).

A clear conclusion from this systematic review is that the field would benefit from a battery of implicit and explicit MI tasks being conducted across the same groups of autistic children, adolescents and adults. Recent work in typical populations has highlighted that MI involves many different dimensions. For example, Kraeutner et al. (2020) asked typical participants to perform a variety of MI tasks such as questionnaires, hand laterality and chronometry and conducted a PCA analysis on the data. They observed that tasks loaded onto three main components termed “generation,” “manipulation” and “maintenance” of motor images. Conducting a similar study in autistic and non-autistic individuals would allow greater understanding of whether specific MI processes are functionally equivalently or not, and whether social ability may be related to the different tasks. Similarly, it will also be important to directly compare implicit and explicit MI ability as they are likely to involve different processes: implicit MI mostly relies on motor representations within the parietal cortex and may not require motor preparation or control related to the activation of supplementary motor area usually associated with explicit MI (Hétu et al., 2013). A further aspect that future studies would benefit from is a comparison of MI with execution of the same action, or some measure of motor ability, to assess the potential relationships between motor coordination difficulties and MI. It is possible that autistic participants with motor coordination difficulties may still be able to accurately imagine their own (less accurate or slower) movements as has been found for people with Parkinson’s Disease (for discussion of this see, Poliakoff, 2013).

A better understanding of MI in autistic individuals has implications around support for motor coordination difficulties that are common in this population but infrequently assessed or treated (Zampella et al., 2021). Research is beginning to highlight the benefit of using MI to improve motor coordination in individuals with Developmental Coordination Disorder (Scott et al., 2021), suggesting that this may also be a useful approach for autistic people. Using qualitative focus group methods, Gowen et al. (2023) recently described the range of motor coordination difficulties and their impact from the viewpoint of autistic adults. They also explored what strategies participants used and some participants described the importance of pre-planning, visualization and imagining, although others found this too challenging. Therefore, employing MI techniques may well be beneficial for some autistic people, although an individual approach is likely to be most appropriate. Indeed, MI ability in typical populations is variable (Moran et al., 2012; Cumming and Eaves, 2018) and influenced by multiple factors such as sporting and dance experience, MI practice, motor and cognitive ability and tactile discrimination (Isaac and Marks, 1994; Arvinen-Barrow et al., 2007; Pelletier et al., 2018; Krüger et al., 2020; Dhouibi et al., 2021; Mao et al., 2022). Therefore, future research examining MI ability and impact of MI training on motor coordination in autistic populations will need to take into account individual differences.

## Limitations

One limitation of the literature on MI in autism is the presence of a sex bias with most of the studies mainly including males. Kessler and Wang (2012) showed that females are more likely to take an embodied approach (i.e., imagining oneself in the new orientation) to visual perspective tasks, whereas other studies suggest that males are better than females on mental rotation (Geiser et al., 2008). It will be important for future studies to include more balanced groups and examine the presence of possible sex differences. A further limitation is that the majority of studies involving the hand laterality task involve children, making it difficult to generalize the findings to the adult population. Explicit MI tasks were too varied and few to draw firm conclusions. A third limitation was the lack of studies investigating the neural processes underlying MI in autistic individuals. This would be a valuable future direction, particularly in relation to identifying possible differences in *how* autistic individuals might perform MI tasks. Finally, one study was included that was labeled as “exclude” according to quality assessment, but we felt it was important to present the study due to this being the first systematic review of the topic the overall small number of studies on the topic of MI in autism.

## Conclusion

In summary, the presence of a biomechanical effect in autistic children performing hand rotation tasks suggests that they are able to implicitly use MI. However, further research on both hand and body rotation tasks is required in both children and adults to follow up suggestions that there may be group differences in the effort or depth of MI. Conclusions about explicit MI tasks are complicated by the small number of studies, the range of tasks conducted, with some the tasks potentially not tapping into MI. The field would benefit from research comparing a range of MI tasks in autistic and non-autistic children and adults to better understand what elements of MI may be affected and to assess the therapeutic potential of MI in these populations.

## Data availability statement

The original contributions presented in the study are included in the article/Supplementary material, further inquiries can be directed to the corresponding author.

## Author contributions

EG: Conceptualization, Data curation, Investigation, Methodology, Supervision, Writing – original draft, Writing – review & editing. EE: Data curation, Investigation, Methodology, Writing – original draft, Writing – review & editing. EP: Conceptualization, Investigation, Methodology, Writing – review & editing.
