# Supplementary material for: Motor imagery in autism: a systematic review
Source: Front Integr Neurosci. 2024 Feb 12;18:1335694. doi: 10.3389/fnint.2024.1335694 (PMC10895877; doi:10.3389/fnint.2024.1335694)
Supplement: Supplementary file 3 [file Table_3.docx]

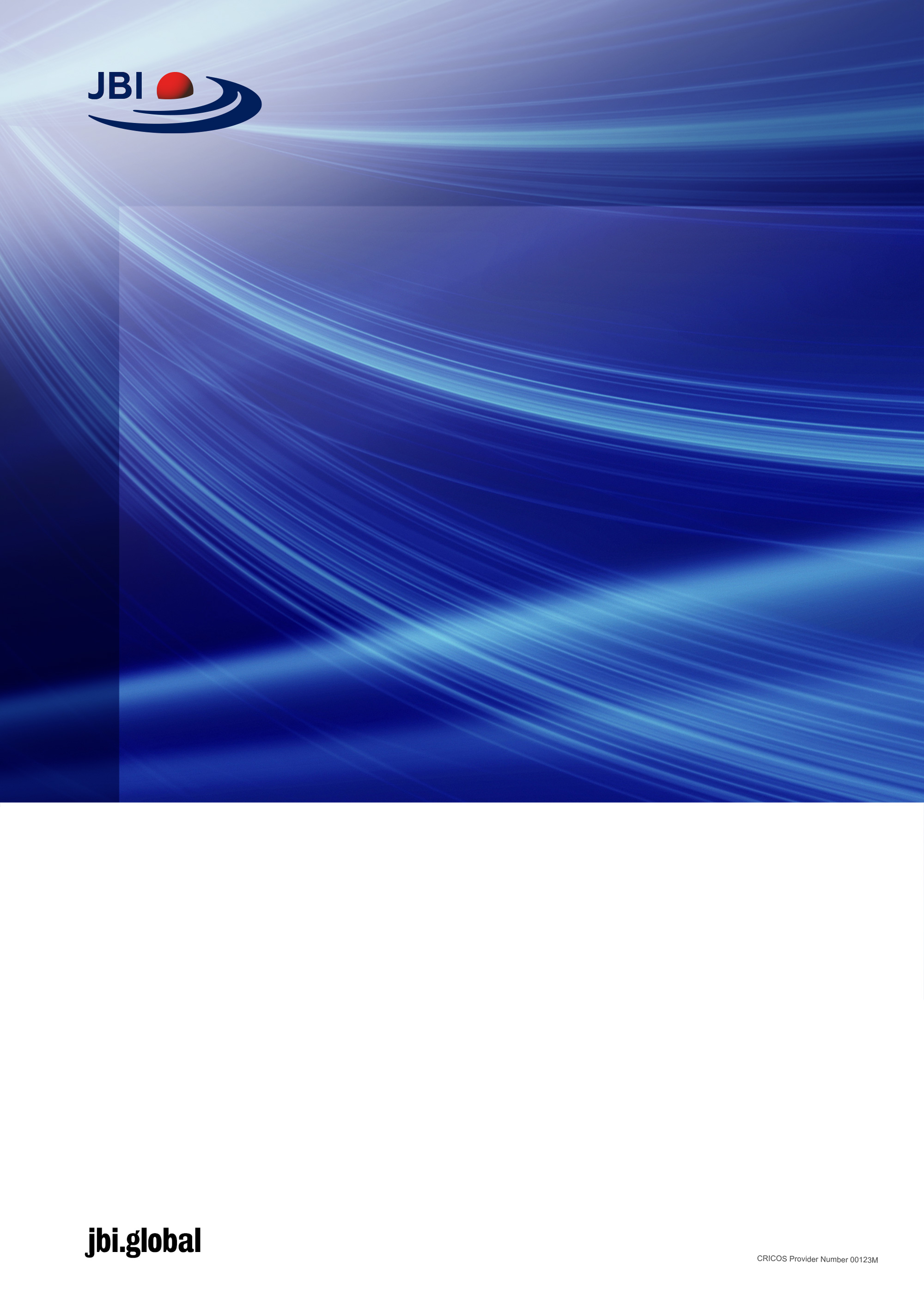


checklist for case control studies

Critical Appraisal tools for use in JBI Systematic Reviews

Introduction

JBI is an international research organisation based in the Faculty of Health and Medical Sciences at the University of Adelaide, South Australia. JBI develops and delivers unique evidence-based information, software, education and training designed to improve healthcare practice and health outcomes. With over 70 Collaborating Entities, servicing over 90 countries, JBI is a recognised global leader in evidence-based healthcare.

## JBI Systematic Reviews

The core of evidence synthesis is the systematic review of literature of a particular intervention, condition or issue. The systematic review is essentially an analysis of the available literature (that is, evidence) and a judgment of the effectiveness or otherwise of a practice, involving a series of complex steps. JBI takes a particular view on what counts as evidence and the methods utilised to synthesise those different types of evidence. In line with this broader view of evidence, JBI has developed theories, methodologies and rigorous processes for the critical appraisal and synthesis of these diverse forms of evidence in order to aid in clinical decision-making in healthcare. There now exists JBI guidance for conducting reviews of effectiveness research, qualitative research, prevalence/incidence, etiology/risk, economic evaluations, text/opinion, diagnostic test accuracy, mixed-methods, umbrella reviews and scoping reviews. Further information regarding JBI systematic reviews can be found in the [JBI Evidence Synthesis Manual](https://jbi-global-wiki.refined.site/space/MANUAL).

## JBI Critical Appraisal Tools

All systematic reviews incorporate a process of critique or appraisal of the research evidence. The purpose of this appraisal is to assess the methodological quality of a study and to determine the extent to which a study has addressed the possibility of bias in its design, conduct and analysis. All papers selected for inclusion in the systematic review (that is – those that meet the inclusion criteria described in the protocol) need to be subjected to rigorous appraisal by two critical appraisers. The results of this appraisal can then be used to inform synthesis and interpretation of the results of the study. JBI Critical appraisal tools have been developed by the JBI and collaborators and approved by the JBI Scientific Committee following extensive peer review. Although designed for use in systematic reviews, JBI critical appraisal tools can also be used when creating Critically Appraised Topics (CAT), in journal clubs and as an educational tool.

JBI Critical Appraisal Checklist for
case control studies

Reviewer__Emma Gowen_______________________ Date_20/10/23______________________________

Author__________Chen et al. __________________ Year___2018___ Record Number_________

|  | Yes | No | Unclear | Not applicable |
| --- | --- | --- | --- | --- |
| 1. Were the groups comparable other than the presence of disease in cases or the absence of disease in controls? | X | □ | □ | □ |
| 1. Were cases and controls matched appropriately? | □ | □ | X | □ |
| 1. Were the same criteria used for identification of cases and controls? | □ | □ | □ | X |
| 1. Was exposure measured in a standard, valid and reliable way? | X | □ | □ | □ |
| 1. Was exposure measured in the same way for cases and controls? | X | □ | □ | □ |
| 1. Were confounding factors identified? | X | □ | □ | □ |
| 1. Were strategies to deal with confounding factors stated? | X | □ | □ | □ |
| 1. Were outcomes assessed in a standard, valid and reliable way for cases and controls? | X | □ | □ | □ |
| 1. Was the exposure period of interest long enough to be meaningful? | □ | □ | □ | X |
| 1. Was appropriate statistical analysis used? | X | □ | □ | □ |

Overall appraisal: Include X Exclude □ Seek further info □

Comments (Including reason for exclusion)

________________________________________________________________________________________________________________________________________________________________________________________________________________________________________________________________________________________________

JBI Critical Appraisal Checklist for
case control studies

Reviewer__Emma Gowen_______________________ Date_20/10/23______________________________

Author__________Conson et al. __________________ Year___2016___ Record Number_________

|  | Yes | No | Unclear | Not applicable |
| --- | --- | --- | --- | --- |
| 1. Were the groups comparable other than the presence of disease in cases or the absence of disease in controls? | X | □ | □ | □ |
| 1. Were cases and controls matched appropriately? | □ | □ | X | □ |
| 1. Were the same criteria used for identification of cases and controls? | □ | □ | □ | X |
| 1. Was exposure measured in a standard, valid and reliable way? | X | □ | □ | □ |
| 1. Was exposure measured in the same way for cases and controls? | X | □ | □ | □ |
| 1. Were confounding factors identified? | X | □ | □ | □ |
| 1. Were strategies to deal with confounding factors stated? | X | □ | □ | □ |
| 1. Were outcomes assessed in a standard, valid and reliable way for cases and controls? | X | □ | □ | □ |
| 1. Was the exposure period of interest long enough to be meaningful? | □ | □ | □ | X |
| 1. Was appropriate statistical analysis used? | X | □ | □ | □ |

Overall appraisal: Include X Exclude □ Seek further info □

Comments (Including reason for exclusion)

______Some, but not all confounding factors identified __________________________________________________________________________________________________________________________________________________________________________________________________________________________________________________________________________________________

JBI Critical Appraisal Checklist for
case control studies

Reviewer__Emma Gowen_______________________ Date_20/10/23______________________________

Author__________Conson et al. __________________ Year___2013___ Record Number_________

|  | Yes | No | Unclear | Not applicable |
| --- | --- | --- | --- | --- |
| 1. Were the groups comparable other than the presence of disease in cases or the absence of disease in controls? | X | □ | □ | □ |
| 1. Were cases and controls matched appropriately? | □ | □ | X | □ |
| 1. Were the same criteria used for identification of cases and controls? | □ | □ | □ | X |
| 1. Was exposure measured in a standard, valid and reliable way? | X | □ | □ | □ |
| 1. Was exposure measured in the same way for cases and controls? | X | □ | □ | □ |
| 1. Were confounding factors identified? | X | □ | □ | □ |
| 1. Were strategies to deal with confounding factors stated? | X | □ | □ | □ |
| 1. Were outcomes assessed in a standard, valid and reliable way for cases and controls? | X | □ | □ | □ |
| 1. Was the exposure period of interest long enough to be meaningful? | □ | □ | □ | X |
| 1. Was appropriate statistical analysis used? | X | □ | □ | □ |

Overall appraisal: Include X Exclude □ Seek further info □

Comments (Including reason for exclusion)

Some, but not all confounding factors identified ___________________________________________________________________________________________________________________________________________________________________________________________________________________________________________________________________________________________

JBI Critical Appraisal Checklist for
case control studies

Reviewer__Emma Gowen_______________________ Date_20/10/23______________________________

Author__Conson et al. [body rotation and perspective taking task]___Year___2015___ Record Number_________

|  | Yes | No | Unclear | Not applicable |
| --- | --- | --- | --- | --- |
| 1. Were the groups comparable other than the presence of disease in cases or the absence of disease in controls? | X | □ | □ | □ |
| 1. Were cases and controls matched appropriately? | □ | □ | X | □ |
| 1. Were the same criteria used for identification of cases and controls? | □ | □ | □ | X |
| 1. Was exposure measured in a standard, valid and reliable way? | X | □ | □ | □ |
| 1. Was exposure measured in the same way for cases and controls? | X | □ | □ | □ |
| 1. Were confounding factors identified? | X | □ | □ | □ |
| 1. Were strategies to deal with confounding factors stated? | X | □ | □ | □ |
| 1. Were outcomes assessed in a standard, valid and reliable way for cases and controls? | X | □ | □ | □ |
| 1. Was the exposure period of interest long enough to be meaningful? | □ | □ | □ | X |
| 1. Was appropriate statistical analysis used? | X | □ | □ | □ |

Overall appraisal: Include X Exclude □ Seek further info □

Comments (Including reason for exclusion)

________________ SOME, BUT NOT ALL CONFOUNDING FACTORS IDENTIFIED ________________________________________________________________________________________________________________________________________________________________________________________________________________________________________________________________________________

JBI Critical Appraisal Checklist for
case control studies

Reviewer__Emma Gowen_______________________ Date_20/10/23______________________________

Author__________David et al __________________ Year___2010___ Record Number_________

|  | Yes | No | Unclear | Not applicable |
| --- | --- | --- | --- | --- |
| 1. Were the groups comparable other than the presence of disease in cases or the absence of disease in controls? | X | □ | □ | □ |
| 1. Were cases and controls matched appropriately? | □ | □ | X | □ |
| 1. Were the same criteria used for identification of cases and controls? | □ | □ | □ | X |
| 1. Was exposure measured in a standard, valid and reliable way? | X | □ | □ | □ |
| 1. Was exposure measured in the same way for cases and controls? | X | □ | □ | □ |
| 1. Were confounding factors identified? | X | □ | □ | □ |
| 1. Were strategies to deal with confounding factors stated? | X | □ | □ | □ |
| 1. Were outcomes assessed in a standard, valid and reliable way for cases and controls? | X | □ | □ | □ |
| 1. Was the exposure period of interest long enough to be meaningful? | □ | □ | □ | X |
| 1. Was appropriate statistical analysis used? | X | □ | □ | □ |

Overall appraisal: Include X Exclude □ Seek further info □

Comments (Including reason for exclusion)

________________________________________________________________________________________________________________________________________________________________________________________________________________________________________________________________________________

JBI Critical Appraisal Checklist for
case control studies

Reviewer__Emma Gowen_______________________ Date_20/10/23______________________________

Author__________Gauthier et al __________________ Year___2018___ Record Number_________

|  | Yes | No | Unclear | Not applicable |
| --- | --- | --- | --- | --- |
| 1. Were the groups comparable other than the presence of disease in cases or the absence of disease in controls? | X | □ | □ | □ |
| 1. Were cases and controls matched appropriately? | □ | □ | X | □ |
| 1. Were the same criteria used for identification of cases and controls? | □ | □ | □ | X |
| 1. Was exposure measured in a standard, valid and reliable way? | X | □ | □ | □ |
| 1. Was exposure measured in the same way for cases and controls? | X | □ | □ | □ |
| 1. Were confounding factors identified? | X | □ | □ | □ |
| 1. Were strategies to deal with confounding factors stated? | X | □ | □ | □ |
| 1. Were outcomes assessed in a standard, valid and reliable way for cases and controls? | X | □ | □ | □ |
| 1. Was the exposure period of interest long enough to be meaningful? | □ | □ | □ | X |
| 1. Was appropriate statistical analysis used? | X | □ | □ | □ |

Overall appraisal: Include X Exclude □ Seek further info □

Comments (Including reason for exclusion)

_______________________________________________________________________________________________________________________________________________________________________________________________________________________________________________________________________________

JBI Critical Appraisal Checklist for
case control studies

Reviewer__Emma Gowen_______________________ Date_20/10/23______________________________

Author__________Gowen et al __________________ Year___2022___ Record Number_________

|  | Yes | No | Unclear | Not applicable |
| --- | --- | --- | --- | --- |
| 1. Were the groups comparable other than the presence of disease in cases or the absence of disease in controls? | X | □ | □ | □ |
| 1. Were cases and controls matched appropriately? | □ | □ | X | □ |
| 1. Were the same criteria used for identification of cases and controls? | □ | □ | □ | X |
| 1. Was exposure measured in a standard, valid and reliable way? | X | □ | □ | □ |
| 1. Was exposure measured in the same way for cases and controls? | X | □ | □ | □ |
| 1. Were confounding factors identified? | X | □ | □ | □ |
| 1. Were strategies to deal with confounding factors stated? | X | □ | □ | □ |
| 1. Were outcomes assessed in a standard, valid and reliable way for cases and controls? | X | □ | □ | □ |
| 1. Was the exposure period of interest long enough to be meaningful? | □ | □ | □ | X |
| 1. Was appropriate statistical analysis used? | X | □ | □ | □ |

Overall appraisal: Include X Exclude □ Seek further info □

Comments (Including reason for exclusion)

________________ SOME, BUT NOT ALL CONFOUNDING FACTORS IDENTIFIED ________________________________________________________________________________________________________________________________________________________________________________________________________________________________________________________________________________

JBI Critical Appraisal Checklist for
case control studies

Reviewer__Emma Gowen_______________________ Date_20/10/23______________________________

Author__________Johansson et al. __________________ Year___2022___ Record Number_________

|  | Yes | No | Unclear | Not applicable |
| --- | --- | --- | --- | --- |
| 1. Were the groups comparable other than the presence of disease in cases or the absence of disease in controls? | □ | X | □ | □ |
| 1. Were cases and controls matched appropriately? | X | □ | □ | □ |
| 1. Were the same criteria used for identification of cases and controls? | □ | □ | □ | X |
| 1. Was exposure measured in a standard, valid and reliable way? | X | □ | □ | □ |
| 1. Was exposure measured in the same way for cases and controls? | X | □ | □ | □ |
| 1. Were confounding factors identified? | X | □ | □ | □ |
| 1. Were strategies to deal with confounding factors stated? | X | □ | □ | □ |
| 1. Were outcomes assessed in a standard, valid and reliable way for cases and controls? | X | □ | □ | □ |
| 1. Was the exposure period of interest long enough to be meaningful? | □ | □ | □ | X |
| 1. Was appropriate statistical analysis used? | X | □ | □ | □ |

Overall appraisal: Include X Exclude □ Seek further info □

Comments (Including reason for exclusion)

________________________________________________________________________________________________________________________________________________________________________________________________________________________________________________________________________________________________

JBI Critical Appraisal Checklist for
case control studies

Reviewer__Emma Gowen_______________________ Date_20/10/23______________________________

Author__________Pearson et al. __________________ Year___2016___ Record Number_________

|  | Yes | No | Unclear | Not applicable |
| --- | --- | --- | --- | --- |
| 1. Were the groups comparable other than the presence of disease in cases or the absence of disease in controls? | X | □ | □ | □ |
| 1. Were cases and controls matched appropriately? | □ | X | □ | □ |
| 1. Were the same criteria used for identification of cases and controls? | □ | □ | □ | X |
| 1. Was exposure measured in a standard, valid and reliable way? | X | □ | □ | □ |
| 1. Was exposure measured in the same way for cases and controls? | X | □ | □ | □ |
| 1. Were confounding factors identified? | X | □ | □ | □ |
| 1. Were strategies to deal with confounding factors stated? | X | □ | □ | □ |
| 1. Were outcomes assessed in a standard, valid and reliable way for cases and controls? | X | □ | □ | □ |
| 1. Was the exposure period of interest long enough to be meaningful? | □ | □ | □ | X |
| 1. Was appropriate statistical analysis used? | X | □ | □ | □ |

Overall appraisal: Include X Exclude □ Seek further info □

Comments (Including reason for exclusion)

________________ SOME, BUT NOT ALL CONFOUNDING FACTORS IDENTIFIED ________________________________________________________________________________________________________________________________________________________________________________________________________________________________________________________________________________

JBI Critical Appraisal Checklist for
case control studies

Reviewer__Emma Gowen_______________________ Date_20/10/23______________________________

Author__________Pearson et al. __________________ Year___2014___ Record Number_________

|  | Yes | No | Unclear | Not applicable |
| --- | --- | --- | --- | --- |
| 1. Were the groups comparable other than the presence of disease in cases or the absence of disease in controls? | X | □ | □ | □ |
| 1. Were cases and controls matched appropriately? | X | □ | □ | □ |
| 1. Were the same criteria used for identification of cases and controls? | □ | □ | □ | X |
| 1. Was exposure measured in a standard, valid and reliable way? | X | □ | □ | □ |
| 1. Was exposure measured in the same way for cases and controls? | X | □ | □ | □ |
| 1. Were confounding factors identified? | X | □ | □ | □ |
| 1. Were strategies to deal with confounding factors stated? | X | □ | □ | □ |
| 1. Were outcomes assessed in a standard, valid and reliable way for cases and controls? | X | □ | □ | □ |
| 1. Was the exposure period of interest long enough to be meaningful? | □ | □ | □ | X |
| 1. Was appropriate statistical analysis used? | X | □ | □ | □ |

Overall appraisal: Include X Exclude □ Seek further info □

Comments (Including reason for exclusion)

________________Participant groups not matched on handedness ________________________________________________________________________________________________________________________________________________________________________________________________________________________________________________________________________________

JBI Critical Appraisal Checklist for
case control studies

Reviewer__Emma Gowen_______________________ Date_20/10/23______________________________

Author__________Ring et al __________________ Year___2018___ Record Number_________

|  | Yes | No | Unclear | Not applicable |
| --- | --- | --- | --- | --- |
| 1. Were the groups comparable other than the presence of disease in cases or the absence of disease in controls? | X | □ | □ | □ |
| 1. Were cases and controls matched appropriately? | □ | □ | X | □ |
| 1. Were the same criteria used for identification of cases and controls? | □ | □ | □ | X |
| 1. Was exposure measured in a standard, valid and reliable way? | X | □ | □ | □ |
| 1. Was exposure measured in the same way for cases and controls? | X | □ | □ | □ |
| 1. Were confounding factors identified? | X | □ | □ | □ |
| 1. Were strategies to deal with confounding factors stated? | X | □ | □ | □ |
| 1. Were outcomes assessed in a standard, valid and reliable way for cases and controls? | X | □ | □ | □ |
| 1. Was the exposure period of interest long enough to be meaningful? | □ | □ | □ | X |
| 1. Was appropriate statistical analysis used? | X | □ | □ | □ |

Overall appraisal: Include X Exclude □ Seek further info □

Comments (Including reason for exclusion)

________________ SOME, BUT NOT ALL CONFOUNDING FACTORS IDENTIFIED ________________________________________________________________________________________________________________________________________________________________________________________________________________________________________________________________________________

JBI Critical Appraisal Checklist for
case control studies

Reviewer__Emma Gowen_______________________ Date_20/10/23______________________________

Author__________ Soulieres et. al. __________________ Year___2011___ Record Number_________

|  | Yes | No | Unclear | Not applicable |
| --- | --- | --- | --- | --- |
| 1. Were the groups comparable other than the presence of disease in cases or the absence of disease in controls? | X | □ | □ | □ |
| 1. Were cases and controls matched appropriately? | □ | □ | X | □ |
| 1. Were the same criteria used for identification of cases and controls? | □ | □ | □ | X |
| 1. Was exposure measured in a standard, valid and reliable way? | X | □ | □ | □ |
| 1. Was exposure measured in the same way for cases and controls? | X | □ | □ | □ |
| 1. Were confounding factors identified? | X | □ | □ | □ |
| 1. Were strategies to deal with confounding factors stated? | X | □ | □ | □ |
| 1. Were outcomes assessed in a standard, valid and reliable way for cases and controls? | X | □ | □ | □ |
| 1. Was the exposure period of interest long enough to be meaningful? | □ | □ | □ | X |
| 1. Was appropriate statistical analysis used? | X | □ | □ | □ |

Overall appraisal: Include X Exclude □ Seek further info □

Comments (Including reason for exclusion)

____ SOME, BUT NOT ALL CONFOUNDING FACTORS IDENTIFIED ___________________________________________________________________________________________________________________________________________________________

JBI Critical Appraisal Checklist for
case control studies

Reviewer__Emma Gowen_______________________ Date_20/10/23______________________________

Author__________Xie et al [recall task]__________________ Year___2018___ Record Number_________

|  | Yes | No | Unclear | Not applicable |
| --- | --- | --- | --- | --- |
| 1. Were the groups comparable other than the presence of disease in cases or the absence of disease in controls? | X | □ | □ | □ |
| 1. Were cases and controls matched appropriately? | □ | □ | X | □ |
| 1. Were the same criteria used for identification of cases and controls? | □ | □ | □ | X |
| 1. Was exposure measured in a standard, valid and reliable way? | X | □ | □ | □ |
| 1. Was exposure measured in the same way for cases and controls? | X | □ | □ | □ |
| 1. Were confounding factors identified? | X | □ | □ | □ |
| 1. Were strategies to deal with confounding factors stated? | X | □ | □ | □ |
| 1. Were outcomes assessed in a standard, valid and reliable way for cases and controls? | X | □ | □ | □ |
| 1. Was the exposure period of interest long enough to be meaningful? | □ | □ | □ | X |
| 1. Was appropriate statistical analysis used? | X | □ | □ | □ |

Overall appraisal: Include X Exclude □ Seek further info □

Comments (Including reason for exclusion)

_______________________________________________________________________________________________________________________________________________________________________________________________________________________________________________________________________________

JBI Critical Appraisal Checklist for
case control studies

Reviewer__Emma Gowen_______________________ Date_20/10/23______________________________

Author__________Xie et al. _[hand rotation task]___________ Year___2022___ Record Number_________

|  | Yes | No | Unclear | Not applicable |
| --- | --- | --- | --- | --- |
| 1. Were the groups comparable other than the presence of disease in cases or the absence of disease in controls? | □ | □ | X | □ |
| 1. Were cases and controls matched appropriately? | □ | □ | X | □ |
| 1. Were the same criteria used for identification of cases and controls? | □ | □ | □ | X |
| 1. Was exposure measured in a standard, valid and reliable way? | □ | □ | X | □ |
| 1. Was exposure measured in the same way for cases and controls? | □ | □ | X | □ |
| 1. Were confounding factors identified? | □ | X | □ | □ |
| 1. Were strategies to deal with confounding factors stated? | □ | X | □ | □ |
| 1. Were outcomes assessed in a standard, valid and reliable way for cases and controls? | □ | X | □ | □ |
| 1. Was the exposure period of interest long enough to be meaningful? | □ | □ | □ | X |
| 1. Was appropriate statistical analysis used? | □ | □ | X | □ |

Overall appraisal: Include □ Exclude X Seek further info □

Comments (Including reason for exclusion)

________________Limited methodological details as supplementary task to main study. ________________________________________________________________________________________________________________________________________________________________________________________________________________________________________________________________________________

Explanation of case control studies
critical appraisal

How to cite: *Moola S, Munn Z, Tufanaru C, Aromataris E, Sears K, Sfetcu R, Currie M, Qureshi R, Mattis P, Lisy K, Mu P-F. Chapter 7: Systematic reviews of etiology and risk . In: Aromataris E, Munn Z (Editors)*. JBI Manual for Evidence Synthesis. JBI, 2020. Available from <https://synthesismanual.jbi.global>

**Case Control Studies Critical Appraisal Tool**

Answers: Yes, No, Unclear or Not/Applicable

## Were the groups comparable other than presence of disease in cases or absence of disease in controls?

The control group should be representative of the source population that produced the cases. This is usually done by individual matching; wherein controls are selected for each case on the basis of similarity with respect to certain characteristics other than the exposure of interest. Frequency or group matching is an alternative method. Selection bias may result if the groups are not comparable.

## Were cases and controls matched appropriately?

As in item 1, the study should include clear definitions of the source population. Sources from which cases and controls were recruited should be carefully looked at. For example, cancer registries may be used to recruit participants in a study examining risk factors for lung cancer, which typify population-based case control studies. Study participants may be selected from the target population, the source population, or from a pool of eligible participants (such as in hospital-based case control studies).

## Were the same criteria used for identification of cases and controls?

It is useful to determine if patients were included in the study based on either a specified diagnosis or definition. This is more likely to decrease the risk of bias. Characteristics are another useful approach to matching groups, and studies that did not use specified diagnostic methods or definitions should provide evidence on matching by key characteristics. A case should be defined clearly. It is also important that controls must fulfil all the eligibility criteria defined for the cases except for those relating to diagnosis of the disease.

## Was exposure measured in a standard, valid and reliable way?

The study should clearly describe the method of measurement of exposure. Assessing validity requires that a 'gold standard' is available to which the measure can be compared. The validity of exposure measurement usually relates to whether a current measure is appropriate or whether a measure of past exposure is needed.

Case control studies may investigate many different ‘exposures’ that may or may not be associated with the condition. In these cases, reviewers should use the main exposure of interest for their review to answer this question when using this tool at the study level.

Reliability refers to the processes included in an epidemiological study to check repeatability of measurements of the exposures. These usually include intra-observer reliability and inter-observer reliability.

[Author note: Exposure was taken to mean the delivery of the task]

## Was exposure measured in the same way for cases and controls?

As in item 4, the study should clearly describe the method of measurement of exposure. The exposure measures should be clearly defined and described in detail. Assessment of exposure or risk factors should have been carried out according to same procedures or protocols for both cases and controls.

[Author note: Exposure was taken to mean the delivery of the task]

## Were confounding factors identified?

Confounding has occurred where the estimated intervention exposure effect is biased by the presence of some difference between the comparison groups (apart from the exposure investigated/of interest). Typical confounders include baseline characteristics, prognostic factors, or concomitant exposures (e.g. smoking). A confounder is a difference between the comparison groups and it influences the direction of the study results. A high quality study at the level of case control design will identify the potential confounders and measure them (where possible). This is difficult for studies where behavioral, attitudinal or lifestyle factors may impact on the results.

[Author note: Confounding factors included aspects such as participant characteristics, stimuli characteristics, chosen measures]

## Were strategies to deal with confounding factors stated?

Strategies to deal with effects of confounding factors may be dealt within the study design or in data analysis. By matching or stratifying sampling of participants, effects of confounding factors can be adjusted for. When dealing with adjustment in data analysis, assess the statistics used in the study. Most will be some form of multivariate regression analysis to account for the confounding factors measured. Look out for a description of statistical methods as regression methods such as logistic regression are usually employed to deal with confounding factors/ variables of interest.

[Author note: If confounding factors were raised in the discussion, “Yes” was ticked.]

## Were outcomes assessed in a standard, valid and reliable way for cases and controls?

Read the methods section of the paper. If for e.g. lung cancer is assessed based on existing definitions or diagnostic criteria, then the answer to this question is likely to be yes. If lung cancer is assessed using observer reported, or self-reported scales, the risk of over- or under-reporting is increased, and objectivity is compromised. Importantly, determine if the measurement tools used were validated instruments as this has a significant impact on outcome assessment validity.

Having established the objectivity of the outcome measurement (e.g. lung cancer) instrument, it’s important to establish how the measurement was conducted. Were those involved in collecting data trained or educated in the use of the instrument/s? (e.g. radiographers). If there was more than one data collector, were they similar in terms of level of education, clinical or research experience, or level of responsibility in the piece of research being appraised?

## Was the exposure period of interest long enough to be meaningful?

It is particularly important in a case control study that the exposure time was sufficient enough to show an association between the exposure and the outcome. It may be that the exposure period may be too short or too long to influence the outcome.

## Was appropriate statistical analysis used?

As with any consideration of statistical analysis, consideration should be given to whether there was a more appropriate alternate statistical method that could have been used. The methods section should be detailed enough for reviewers to identify which analytical techniques were used (in particular, regression or stratification) and how specific confounders were measured.

For studies utilizing regression analysis, it is useful to identify if the study identified which variables were included and how they related to the outcome. If stratification was the analytical approach used, were the strata of analysis defined by the specified variables? Additionally, it is also important to assess the appropriateness of the analytical strategy in terms of the assumptions associated with the approach as differing methods of analysis are based on differing assumptions about the data and how it will respond.
